# Plant-based (vegan) diets for pets: A survey of pet owner attitudes and feeding practices

**DOI:** 10.1371/journal.pone.0210806

**Published:** 2019-01-15

**Authors:** Sarah A. S. Dodd, Nick J. Cave, Jennifer L. Adolphe, Anna K. Shoveller, Adronie Verbrugghe

**Affiliations:** 1 Department of Clinical Studies, Ontario Veterinary College, University of Guelph, Guelph, Ontario, Canada; 2 School of Veterinary Science, Massey University, Palmerston North, New Zealand; 3 Petcurean Pet Nutrition, Chilliwack, British Columbia, Canada; 4 Department of Animal Biosciences, University of Guelph, Guelph, Ontario, Canada; Texas A&M University College Station, UNITED STATES

## Abstract

People who avoid eating animals tend to share their homes with animal companions, and moral dilemma may arise when they are faced with feeding animal products to their omnivorous dogs and carnivorous cats. One option to alleviate this conflict is to feed pets a diet devoid of animal ingredients—a ‘plant-based’ or ‘vegan’ diet. The number of pet owners who avoid animal products, either in their own or in their pets’ diet, is not currently known. The objective of this study was to estimate the number of meat-avoiding pet owners, identify concerns regarding conventional animal- and plant-based pet food, and estimate the number of pets fed a plant-based diet. A questionnaire was disseminated online to English-speaking pet owners (n = 3,673) to collect data regarding pet owner demographics, diet, pet type, pet diet, and concerns regarding pet foods. Results found that pet owners were more likely to be vegetarian (6.2%; 229/3,673) or vegan (5.8%; 212/3,673) than previously reported for members of the general population. With the exception of one dog owned by a vegetarian, vegans were the only pet owners who fed plant-based diets to their pets (1.6%; 59/3,673). Of the pet owners who did not currently feed plant-based diets but expressed interest in doing so, a large proportion (45%; 269/599) desired more information demonstrating the nutritional adequacy of plant-based diets. Amongst all pet owners, the concern most commonly reported regarding meat-based pet foods was for the welfare of farm animals (39%; 1,275/3,231). The most common concern regarding strictly plant-based pet foods was regarding the nutritional completeness of the diet (74%; 2,439/3,318). Amongst vegans, factors which predicted the feeding of plant-based diets to their pets were concern regarding the cost of plant-based diets, a lack of concern regarding plant-based diets being unnatural, and reporting no concern at all regarding plant-based diets for pets. Given these findings, further research is warranted to investigate plant-based nutrition for domestic dogs and cats.

## Introduction

Human diets minimising (‘ovo-lacto vegetarian’, ‘vegetarian’) or eschewing animal products completely (‘strict vegetarian’, ‘vegan’) have been increasing in prevalence worldwide in the past decade [1–3]. Motivations reported for this shift away from animal products include health concerns, sustainability/environmental preservation and empathy for non-human animals [4–6]. These motivations are certainly not unfounded. The many health risks associated with human consumption of animal products, including coronary heart disease, stroke, type 2 diabetes, obesity, and a variety of cancers, are becoming widely appreciated by the scientific community and general public [7–12]. The environmental impacts known to be associated with our dietary choices are similarly well documented [13–15], and people are also becoming increasingly concerned about the environmental impact of modern pet foods as well [16]. Traditionally, commercial pet foods were derived mostly from animal and plant by-products from the human food industry, which has been considered a highly sustainable practice [17–19]. However, with many pet owners now seeking by-product-free and meat-centric foods, there may be direct competition with animal products otherwise destined for the human food chain [17, 18]. With regards to empathy for animals, people who have chosen to abstain from eating animals have been reported to possess stronger empathy for non-human species than people who consume meat [20, 21].

Pet owners have also been found to have higher levels of empathy for animals than people who do not live with pets [22, 23]. Considering their high regard for animals, pet owners clearly want to feed diets that they consider healthy and beneficial for their companions’ wellbeing [24–26]. For some, however, this may conflict with ideologies to minimize or avoid use of products derived from other animals. Termed “the vegetarian’s dilemma”, a moral conflict has been documented amongst pet owners who avoid animal products in their own diets and report feelings of guilt and internal conflict regarding feeding animal products to their pets [27]. Recently, vegetarian and plant-based diets have been introduced to the pet food market and provide a potential resolution to this moral dilemma for vegetarians and vegans who share their homes with omnivorous and carnivorous pets [28–31]. Additionally, in one study, over one quarter of vegetarians indicated religion as a primary reason for avoiding meat in their diet [32]. Thus, social considerations, such as cultural or religious practices, may also necessitate avoidance of animal products in pet food. Examples of religions which promote avoidance of meat and/or animal products include Buddhism, Seventh-Day Adventism, and Hare Krishna [32,33].

Considering the facultative and obligatory carnivorous physiology of dogs and cats respectively, the suitability of these plant-based diets in meeting the nutritional needs of these animals has been questioned [30, 34, 35]. Few studies have evaluated the nutritional content of plant-based pet foods or health parameters and nutrient status of pets fed plant-based diets. Neither the nutritional adequacy of commercially available plant-based pet foods nor the health effects on the pets eating those foods are well defined. While dietary avoidance of animal products may confer some benefit to human health, it does so at the risk of insufficiency of essential nutrients, such as vitamins B12 and D, calcium, iodine or zinc [36–38]. Indeed, risk of bone fracture has been reported to be higher amongst vegans than other diet groups, and a case of hypothyroidism has been reported in a child, due to a vegan diet lacking adequate iodine [12, 39]. No evidence for health benefits of meat-avoidance in pet dog or cat diets has been published, and, as with humans, risks of dietary insufficiency are present. Despite concerns of nutritional adequacy, it has been found that pet owners abstaining from eating meat for ethical reasons are significantly less likely to feed their pets diets containing animal products than meat-eating pet owners, with 21% of vegan pet owners and 5% of vegetarian pet owners reporting feeding a diet composed of less than 25% animal products [27]. It is unknown how many pet owners currently feed plant-based diets to their dogs or cats.

Previous surveys have examined feeding practices and attitudes amongst vegetarian and vegan pet owners [27, 28, 40], but these were specifically directed at vegetarians and vegan groups and thus gave no indication as to the scope of the issue amongst the pet population in general. It was therefore the purpose of this study to estimate the number of pet owners who feed plant-based diets to their dogs and/or cats, identify their concerns regarding animal products and ascertain their motivations for feeding plant-based diets. Estimation of the prevalence of this type of feeding provides a baseline from which potential growth of the trend could be measured, as well as to inform veterinarians, nutritionists and pet professionals as to the scope of this controversial practice at present. Understanding the attitudes, concerns and motivations of pet owners feeding their pets plant-based diets is necessary for effective communication between veterinarians and their clients in order to serve both their clients’ and patients’ best interests. Furthermore, the results of this study could provide insight into evolving feeding practices and human-animal relationships and support further investigation in plant-based pet nutrition. It was hypothesized that there would be a higher prevalence of vegetarianism and veganism amongst pet owners than has been reported in the general population, and that there would be more concern regarding animal-derived ingredients in pet foods amongst vegetarians and vegans than other diet groups. It was also hypothesized that vegetarian and vegan pet owners would have fewer concerns regarding plant-based pet foods and would be more likely to feed these diets to their dogs and/or cats as compared to omnivorous pet owners. A high prevalence of meat-avoidance and concern regarding animal-based pet foods may indicate demand for development of nutritionally complete and balanced plant-based diets for pets in order to fulfill pets’ nutritional requirements while simultaneously meeting the needs of their owners.

## Materials and methods

A multiple choice and short answer survey titled “Pet Feeding Practices” was administered online (www.surveymonkey.net). To minimize selection bias, no reference was made to any particular type of diet or feeding practice in the title or introduction of the survey. The study was supported by the research ethics board of the University of Guelph (REB #17-08-029). The questionnaire started with multiple-choice questions [MCQ] collecting demographic data (age, gender, country), pet owner diet (omnivore, pescetarian, vegetarian or vegan, with descriptions of each diet) and pet type (cat, dog or both). An omnivorous diet was defined as one that included meat; pescetarian was defined as including fish but no other meats; vegetarian was defined as avoiding all meat but including eggs, dairy and/or honey; and vegan was defined as being devoid of all animal products. The next series of MCQs also included an open-text option of ‘other’ where the pet owner could input their response. These included locations where pet food was sourced (supermarket, pet store, veterinary clinic, direct from manufacturer, online, homemade, ‘other’) type of pet diet, treats/snacks/scraps fed in addition to the pet’s diet, and concerns regarding pet foods. Type of pet diet was categorized as commercial or homemade; meat-based, vegetarian, or vegan; raw or cooked; and, in the case of cooked commercial diets, kibbled or canned. While the term ‘meat-based’ is not entirely accurate, as many pet foods utilise animal by-products not conventionally considered ‘meat’, such as offal, blood or bone meal and also typically contain a large amount of plant ingredients, this was utilised within the survey text for simplicity. Similarly, the term ‘vegan’ was utilised to refer to pet foods within the text of the survey in order to be easily identifiable. The meaning, however, of the term ‘vegan’ is defined by The Vegan Society as a lifestyle/philosophy, and not simply a diet [41]. Thus, the phrase ‘plant-based’ is used within this paper to describe pet food devoid of animal products, as it is recognized that neither dogs nor cats are free to choose their lifestyle. Feeding frequency of each type of diet was also determined, with pet owners reporting feeding any type of diet either ‘daily’, ‘often’, ‘infrequently’ or ‘never’. Respondents could choose from a list of possible concerns regarding meat-based pet foods: farm animal welfare, farm animal rights, health concerns, environmental concerns, social concerns, and ‘other’; and plant-based pet foods: unnatural, unhealthy, not nutritionally complete and balanced, cost, moral acceptability, and ‘other’. When ‘other’ concerns reported fell within one of the listed categories, these were adjusted in the dataset. For example, mention of cultural or religious practices were included in the ‘social’ concerns category, while concern regarding origin or quality of ingredients was included in the health-related categories. Examples of ‘other’ concerns which were kept within the ‘other’ category include: concerns regarding palatability and unawareness or unavailability of different diet options. For pet owners who indicated that they did not feed a plant-based diet, an extra MCQ was included to ascertain whether they would feed a plant-based diet if one were available that met their standards or desired attributes. For those who indicated they would not feed a plant-based diet even if it met their specific stipulations, a follow-up open-text option question was then asked to differentiate between individuals harbouring further concerns regarding plant-based diets and rejection of the diet based on non-cognitive negative affective response [42]. The survey ended with a set of questions to assess financial motivation of pet owners to feed a commercial plant-based diet, if one were available to them. For pet owners with both cats and dogs, questions were first answered regarding their dog, then their cat, and then finished with the generic questions regarding purchasing of plant-based diets. The questionnaire was piloted on 15 volunteers and questions adjusted as required to improve survey.

The questionnaire was distributed online through social media to a number of pet-centric groups, including dog and cat breeders, owners, and general enthusiasts. Though many of the groups were international, the survey was available only in English. Sharing of the survey was encouraged in order to reach a broad audience. Veterinary hospitals/clinics, other pet professional and para-professional groups, and nutrition-related groups were avoided to minimize selection bias, as the aim of the survey was to collect data from a sample representative of the general pet-owning population. The survey was available online for a total of 135 days, although it was only actively promoted for the first 65 days.

Survey responses were analysed and descriptive statistics used to assess categorical data. Quantitative and qualitative data were reported as frequency (n) and percentage (%). When comparing responses by country, only those countries from which more than 100 responses were received were considered large enough for comparison. Comparison of proportions between the current study and previous studies was performed by calculating 95% confidence intervals. Data collected was categorical. Univariate comparisons were conducted using Chi-square analysis. Multivariable binary logistic regression modeling was used to assess the relationship between variables of interest and feeding practice outcomes. Input variables evaluated were: age, gender, country, pet type, and concerns regarding animal- and plant-based diets, while the output variable was feeding a plant-based diet to pets. Colinearity was accounted for by performing stepwise regression to identify relationships between and amongst variables input into the logistic regression model. Both forward and backward stepwise regression were consistent. Odds ratios were calculated by exact conditional logistic regression to determine the association between variables of interest and the outcome of vegans feeding plant-based diets to their pets. Values less than zero were considered protective against the practice, while values greater than zero were considered predictive of the practice. Statistical significance was set at *P* < 0.05; trends were recognized if *P* was > 0.05 but < 0.1. All analyses were performed and all figures created with commercial software (IBM SPSS Statistics Version 24, IBM Corp, North Castle, New York, USA; SAS 9, SAS Institute, Cary, North Carolina, USA).

## Results

A total of 3,718 questionnaires were commenced, of which 3,673 were complete enough to include for statistical analysis. Those not included were due to attrition prior to identifying type of pet. Survey participants comprised 1,871/3,673 (51%) dog owners, 602/3,673 (16%) cat owners, and 1,200/3,673 (33%) owners of both dogs and cats. Pet owner demographics are presented in Table 1. The survey questions were considered independently, so the number of responses for each question differed, thus proportions are representative only for the number of people answering each question. For example, pet owners who had only cats were not included in data analysis regarding dogs.

**Table 1:** Demographic data of pet owners participating in the “Pet Feeding Practices” survey.

Category	Variable	%	n
Gender	Male	6%	215
	Female	92%	3381
	Prefer not to disclose	2%	77
Age (years)	< 20	4%	129
	21–30	26%	946
	31–40	19%	680
	41–50	20%	734
	51–60	20%	725
	61–70	10%	378
	> 70	2%	81
Country	Australia	7%	258
	Canada	11%	405
	Finland	7%	247
	New Zealand	5%	177
	United Kingdom	23%	836
	United States	41%	1485
	Other	7%	265

Total number of survey respondents = 3,673.

**Fig 1:**
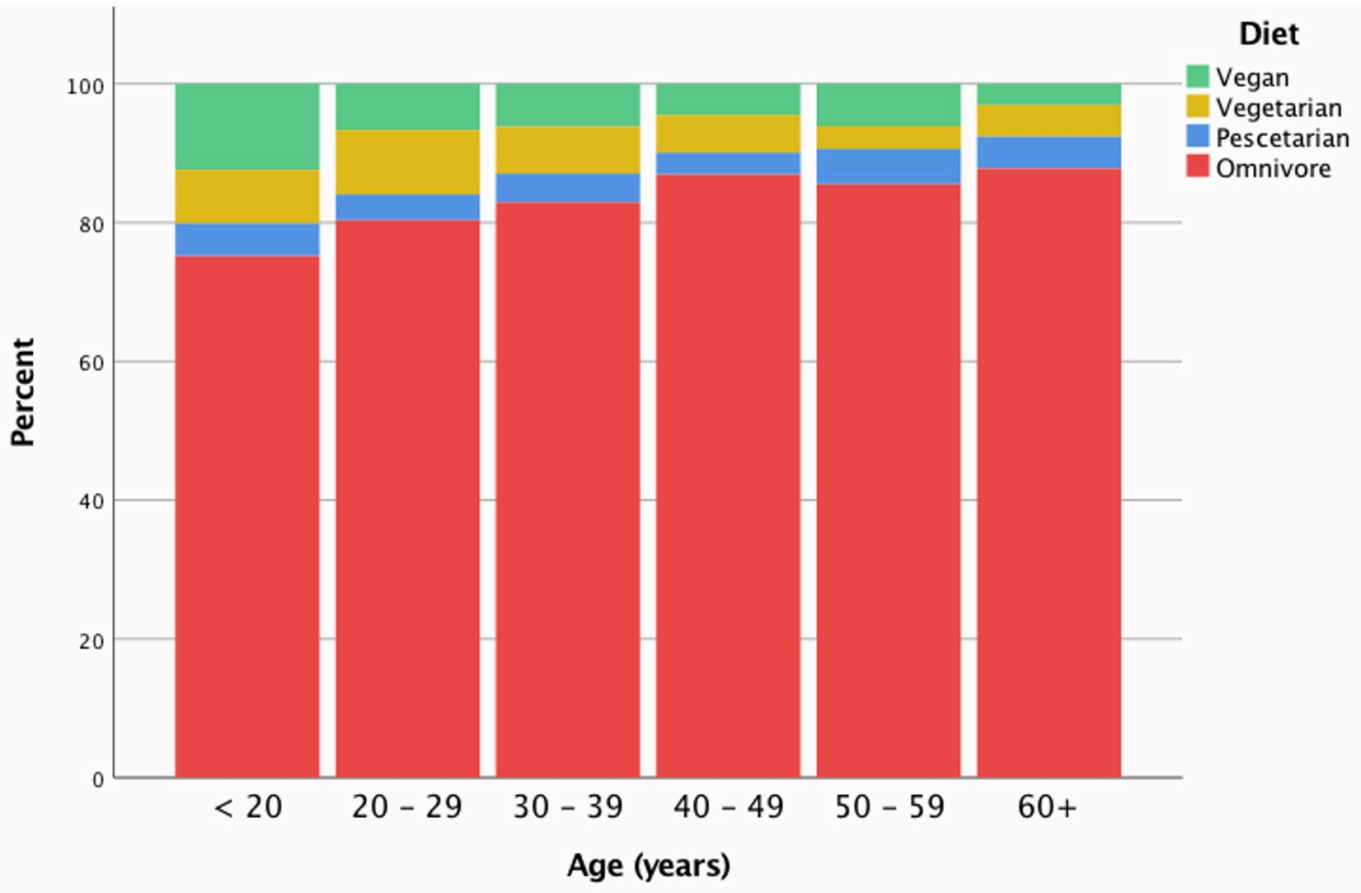
Proportion of pet owners following a given diet, by age group.

The majority of pet owners (84%, 3082/3,673) reported eating an omnivorous diet, with 5.8% (212/3,673) identifying as vegan, 6.2% (229/3,673) as vegetarian, and the remaining 4% (150/3,673) as pescetarian. Differences in meat-avoidance were detected between age groups (*P* = 0.001) and are depicted in Fig 1. Vegetarianism tended to be reported twice as frequently in females as compared to males (3%; 6/215 males vs 6%; 218/3,381 females, *P* = 0.099). A higher prevalence of veganism was reported in males (8.3%, 18/215) than females (5.8% 186/3,381), *P* = 0.046. The prevalence of meat avoidance was different between countries (Table 2).

**Table 2:** Prevalence of veganism and vegetarianism in pet owners participating in the “Pet Feeding Practices” survey compared to the general population of their respective countries.

Country	General population*				Study respondents			
	% Vegetarian		% Vegan		% Vegetarian		% Vegan	
	95% CI	SE	95% CI	SE	95% CI	SE	95% CI	SE
Australia	1.2–2.7	0.004	0–0.2	0.0008	5.5–12.5^a^	0.017	3.9–10.1^a,b^	0.016
Canada	4**	NA	NA	NA	2.8–7.1^a,b^	0.011	8.8–15.2^a^	0.016
Finland	0.35–0.51	0.0004	0.13–0.23	0.0003	6.3–13.7^a^	0.019	4.6–11.4^a,b^	0.017
New Zealand	2.3**	NA	NA	NA	3.2–10.8^a,b^	0.019	0–4.1^b^	0.011
United Kingdom	2.4–3.6	0.003	1.5–2.5	0.002	5.3–8.7^a^	0.009	3.5–6.5^b^	0.008
United States	3.7–6.3	0.007	1.1–2.8	0.004	3.0–5.0^b^	0.005	3.0–5.0^b^	0.005

For data regarding the general population, the symbol ‘NA’ indicates reliable data not available. Significant (Chi Square, *P* < 0.05) differences in vegetarianism and veganism between countries detected in this study are indicated by superscript letter.

*According to other publications [1, 3, 43–53]

**Sample size not recorded, 95% CI cannot be determined

In total, 51% (1,659/3,231) of survey respondents reported at least one concern regarding meat-based pet foods, resulting in a total of 5,115 concerns reported regarding meat-based pet foods. Pet owners over 60 years of age reported fewer concerns (42%; 164/392) than younger age groups (*P* = 0.003). Concerns were more frequently expressed by respondents in the UK (55%; 403/737) and least frequently in the USA (42%; 562/1,326; *P* = 0.001). There were no differences in the frequency of concern expression between different pet categories (dog, cat or both). The most commonly reported concern overall was for the welfare of farmed animals, reported by 39% (1,275/3,231) of pet owners. Significant differences in the number of concerns expressed about animal products in pet foods were detected between pet owners when categorised according to their dietary status. Omnivores reported fewer concerns, and vegans more concerns; vegans also differed from other diet groups in all concern categories except for ‘other’ (Table 3). In comparison, 91% of pet owners (3,044/3,318) reported at least one concern regarding strictly plant-based (‘vegan’) pet foods, for a total of 11,399 concerns reported regarding plant-based pet foods, roughly double that reported for meat-based pet foods. Whereas just over half of pet owners reported concern regarding meat-based pet foods, almost all pet owners indicated concern regarding plant-based pet foods. Differences were detected between age groups, with concerns regarding plant-based pet foods being most frequently reported in the youngest two age groups (95%; 921/971), decreasing with age to be lowest in oldest two age groups (89%; 939/1,059; *P* = 0.001). Pet owners who had dogs only reported the lowest number of concerns (88%; 1,498/1,693) while those who had cats only reported the highest number of concerns (95%; 508/537; *P* = 0.001). As with concerns regarding animal products, the greatest differences between concerns regarding plant-based pet foods were detected among pet owner diet groups (Table 3).

**Table 3:** Concerns regarding pet foods and financial motivation to buy plant-based pet food, reported by pet owners participating in the “Pet Feeding Practices” survey.

	Omnivore	Pescetarian	Vegetarian	Vegan
Concern regarding meat-based diets	n = 2,740	n = 123	n = 205	n = 191
Farm animal welfare	33.4 (916)^a^	71.5 (88)^b^	62.9 (129)^b^	74.3 (142)^b^
Farm animal rights	17.5 (480)^a^	43.9 (54)^b^	47.8 (98)^b^	76.4 (146)^c^
Unhealthy	27.0 (740)^a^	40.7 (50)^b,c^	33.7 (69)^a,c^	47.6 (91)^b^
Environment	18.0 (493)^a^	47.2 (58)^b^	36.6 (75)^b^	64.9 (124)^c^
Social	4.4 (121)^a^	12.2 (15)^b^	7.3 (15)^a,b^	30.9 (59)^c^
Other	3.8 (102)	4.1 (5)	5.4 (11)	4.2 (8)
None	45.9 (1,257)^a^	12.2 (15)^b,c^	22.0 (45)^c^	11.0 (21)^b^
Concern regarding plant-based diets	n = 2,765	n = 134	n = 209	n = 189
Unnatural	69.6 (1,925)^a^	62.7 (84)^a^	71.8 (150)^a^	38.1 (72)^b^
Unhealthy	52.0 (1,439)^a^	61.2 (82)^a,c^	62.2 (130)^b^	40.7 (77)^c^
Incomplete	74.5 (2,059)^a^	76.1 (102)^a^	79.4 (166)^a^	59.3 (112)^b^
Cost	13.3 (364)	13.4 (18)	9.6 (20)	16.4 (31)
Immoral	50.7 (1,402)^a^	45.5 (61)^a^	56.5 (118)^a^	27.0 (51)^b^
Other	9.5 (265)^a^	9.7 (13)^a,b^	17.6 (37)^c^	14.1 (27)^b,c^
None	7.8 (216)^a^	11.2 (15)^a^	7.2 (15)^a^	30.7 (58)^b^
Financial motivation to buy a plant-based diet	n = 2,303	n = 116	n = 182	n = 168
“I would not feed my pet a plant-based diet”	75.3 (1,735)^a^	50.9 (59)^b^	57.7 (105)^b^	22.0 (37)^c^
“I would only feed it if it cost less than what I currently feed”	4.9 (112)	4.3 (5)	5.5 (10)	1.8 (3)
“I would feed it if it cost the same as what I currently feed”	17.5 (403)^a^	33.6 (39)^b^	25.3 (46)^a,b^	29.8 (50)^b^
“I would feed it even if it cost more than what I currently feed”	2.3 (53)^a^	11.2 (13)^b^	11.5 (21)^b^	46.4 (78)^c^

Survey respondents are grouped according to diet, and concerns are shown as percentage and number in parentheses. Superscript letters indicate significant (*P* < 0.0083) differences within rows (between diet groups) as determined by the two-sided test of equality for proportions, tests are adjusted for all pairwise comparisons within a row using the Bonferroni correction.

Dietary information was provided for 2,940 dogs and 1,542 cats. Most pets (dogs 97% 2848/2940; cats 99% 1530/1,545) were fed food that contained meat. Daily feeding of conventional kibble was reported for 61% (1,796/2,940) of dogs and 69% (1,064/1,545) of cats, with daily feeding of conventional canned food to 15% (427/2,940) of dogs and 44% (684/1,545) of cats. Many pets were intermittently fed diets vegetarian or plant-based foods (dogs 10.4% 305/2,940; cats 3.3% 51/1,545), but exclusive feeding of plant-based diets was reported only by vegans and one vegetarian. In total, 1.6% (48/2,940) of dogs and 0.7% (11/1,545) of cats were fed a strictly plant-based diet. Of the pets being fed strictly plant-based diets, the majority (dogs 91% 40/44; cats 73% 8/11) were fed a commercial plant-based diet with inclusion of some homemade foods, while 2% (1/44) of dogs 18% (2/11) of cats fed plant-based diets were fed a homemade plant-based diet exclusively.

**Fig 2:**
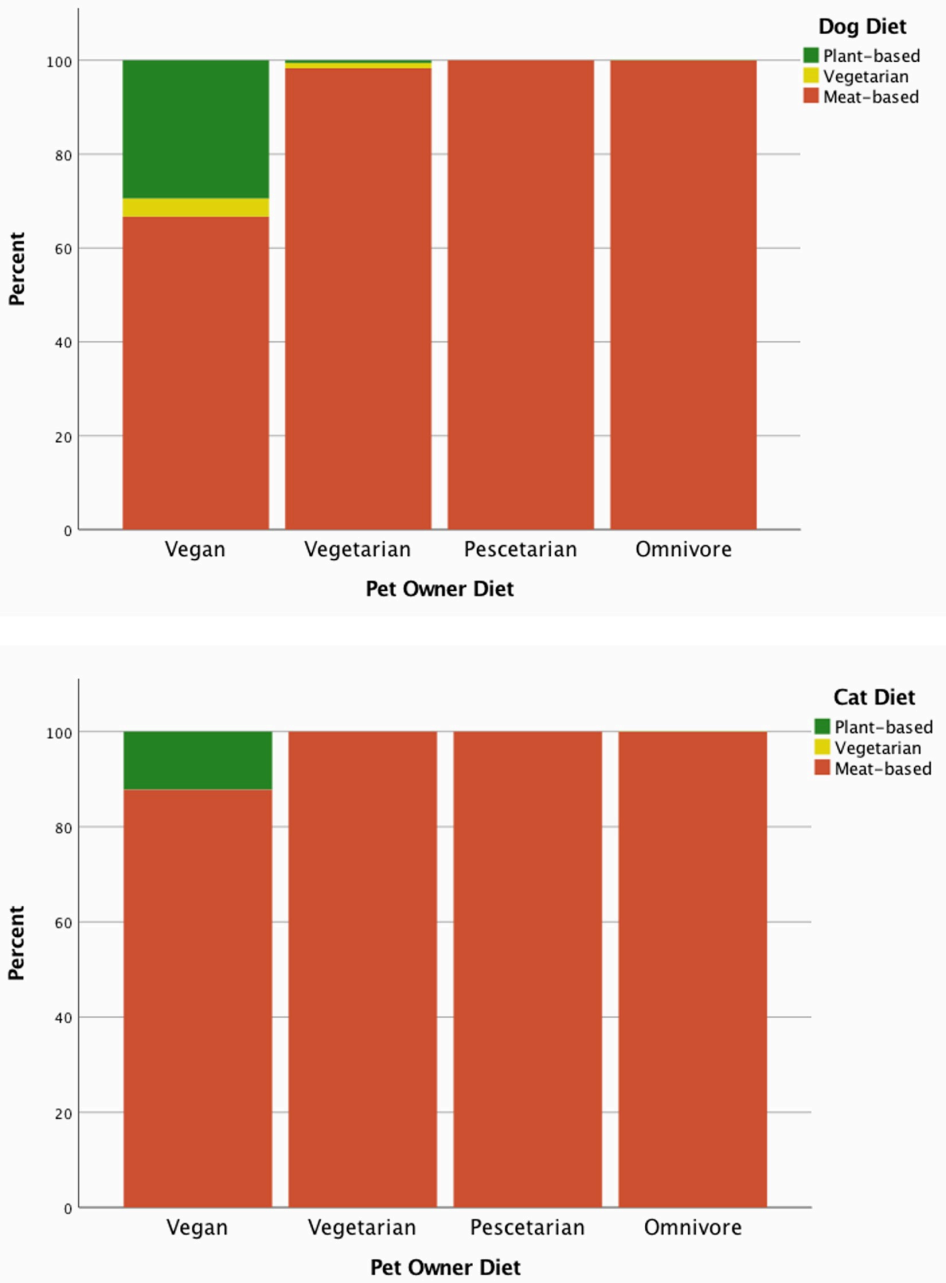
Composition of pet diets according to their owners’ diet. Based on responses of pet owners to the “Pet Feeding Practices” survey, dog top, cat bottom. The Y axis represents percentage of pet owners within a given diet group.

**Fig 3:**
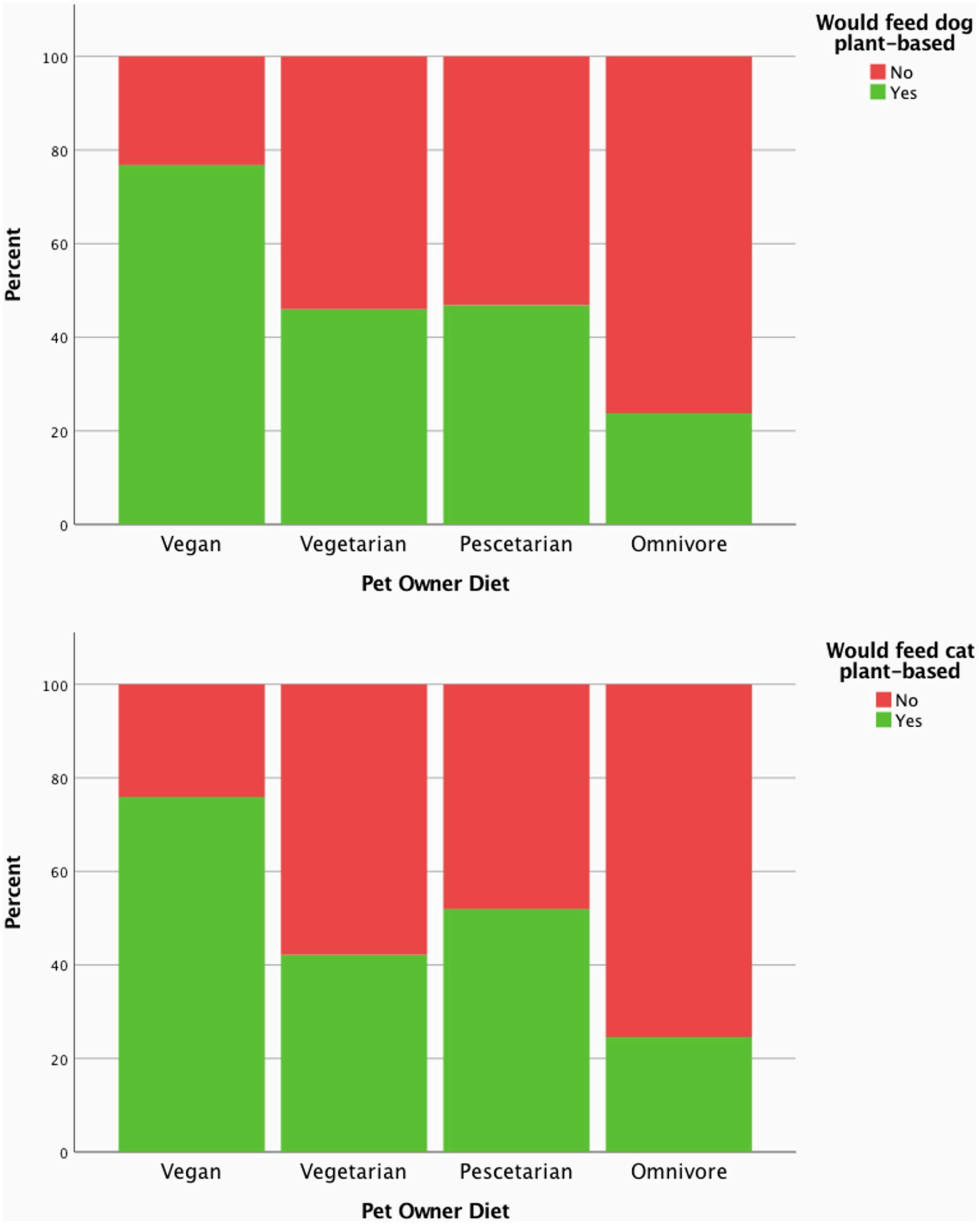
Percentage of pet owners who would consider feeding a plant-based diet if one were available which fit their criteria. Based on responses of pet owners to the “Pet Feeding Practices” survey. Y axis represents percentage of pet owners within a given diet group.

Only 27% (58/212) of vegans reported feeding their pets a plant-based diet, yet 78% (131/168) of vegan pet owners indicated they would feed a plant-based diet to their pet if one were available which met the pet owners’ required criteria (Figs 2 and 3). In total, 35% (1,083/3,130) of pet owners who did not already feed a plant-based diet to their pet indicated interest in doing so, with 55% of those pet owners (599/1,083) stating further stipulations needed to be met before they would do so. Of these pet owners who indicated further stipulations, 45% (269/599) reported a need for further evidence of nutritional sufficiency. Veterinary approval (20%; 122/599) and greater availability (20%; 117/599) were also commonly reported. Motivation to feed a plant-based diet was measured in terms of cost compared to what the pet owner currently paid for their pets’ food. Significant differences in motivation to feed a plant-based diet were detected based on pet owner diets (Table 3). The remainder of pet owners (65%; 1,936/3,130) simply would not feed a plant-based diet, regardless of whether one existed that met all of their criteria.

With the exception of a single vegetarian, only vegan respondents reported feeding a plant-based diet to their pet. Pet owner diet was thus excluded from predictive models, and binary logistic regression was employed to determine what factors were associated with vegan pet owners feeding their pets a plant-based diet. Variables offered initially to the model included: age, gender, pet type, and all concerns reported for animal-based and plant-based diets, the results of which are shown in Table 4. Forward and backward stepwise regression analysis was performed to eliminate non-significant variables and account for inter-variable associations. After stepwise regression, variables found to be most significantly associated with vegan owners feeding their pets a plant-based diet were: concern regarding plant-based diets being unnatural (OR 0.06, *P* = 0.006), concern regarding the cost of plant-based diets (OR 4.78, *P* = 0.009) and no concerns regarding plant-based diets (OR 11.36, *P* <0.001).

**Table 4:** Results of regression model including all variables, showing their associations with vegan pet owners feeding a plant-based diet to their pet based on responses of pet owners to the “Pet Feeding Practices” survey.

Variable	OR	*P* value	95% CI
Age	1.06	.646	0.82–1.38
Gender	1.61	.172	0.82–3.16
Pet type	1.11	.708	0.65–1.91
Concern regarding farm animal welfare	0.42	0.07	0.15–1.09
Concern regarding farm animal rights	5.46	0.04	1.08–27.78
Concern regarding meat-based diets being unhealthy	3.38	0.05	1.45–7.87
Concern regarding the environmental impact of meat-based diets	1.26	.646	0.47–3.38
Concern regarding the social implications of meat-based diets	0.90	.803	0.38–2.12
No concerns regarding meat-based diets	0.00	.971	0.00–1000
Concern regarding plant-based diets being unnatural	0.09	.036	0.01–0.85
Concern regarding plant-based diets being unhealthy	0.71	0.67	0.15–3.40
Concern regarding plant-based diets being nutritionally incomplete	3.08	.198	0.56–16.95
Concern regarding cost of plant-based diets	5.29	.018	1.32–21.28
Concern plant-based diets being immoral	0.00	.951	0.00–1000
No concern regarding plant-based diets	27.78	.001	3.98–200

## Discussion

At this point in time, the diet of most pets included animal products to some degree. However, of those pets fed diets which included animal products, some were regularly fed vegetarian and plant-based foods with occasional animal-derived foods added. Twice as many dogs as cats were fed exclusively plant-based diets. This higher prevalence of plant-based feeding to dogs was not unexpected, considering the more flexible omnivorous physiology of dogs, and the relative lack of commercial plant-based diets for cats. Perhaps counter-intuitively, in this study, meat-avoiding pet owners appeared to keep cats in preference to dogs, though this only reached significance in vegetarians and not vegans. This may be a reflection of some intrinsic preference meat-avoiding pet owners have for cats, particularly since it was found that vegetarians were both more likely to keep cats, and less likely to keep dogs. However, considering the obligatory carnivorous physiology of cats, one may expect pet owners who themselves avoid meat to also avoid having pets who eat meat, though this was not supported by the findings of this study. Other reasons for cat preference among vegetarians may be regarding lifestyle, such as living in a smaller home in an urban setting [54]. Indeed, urbanisation has been found to be correlated with meat avoidance [55]. Other lifestyle factors such as life stage (single vs. couples), size of household, and income may also contribute to the increased prevalence of cat ownership amongst vegetarians. In the USA, cats were preferred over dogs by singles and households with lower income [54]. Similar patterns in terms of life stage, size of household, and household income have also been identified amongst vegetarians, though these factors were not investigated in the current study [55, 56]. Similarly, pescetarians, people following a diet which excludes meat from land animals but includes fish and other marine creatures, who kept cats were found to be more interested in feeding a plant-based diet to their pet than were pescetarians with dogs (Fig 3). It may simply be that the relative dearth of plant-based options for cats leaves more cat owners wanting a suitable alternative, thus inflating the number of those who do not currently feed a plant-based diet but would do so if one were available. Alternatively, this may again represent an association between meat-avoidance and cat-ownership, indicating a possible avenue for future investigation. Regarding the relative lack of commercial plant-based options for cats, comparatively more cats were fed a homemade plant-based diet than reported in dogs, though the sample size was very small. Despite the small number, this finding is particularly concerning, as complete and balanced homemade diets are challenging for pet owners to make even with the inclusion of animal products [25, 57–65], while homemade vegetarian and vegan diets have been considered contraindicated in cats [66].

Logistic regression was performed to predict which factors were associated with vegans choosing to feed a plant-based diet to their pet (Table 4). Vegans pet owners with concerns about plant-based diets being unnatural for their pets were significantly less likely to feed a plant-based diet than those without concern about how natural the diet may be. Similarly, vegan pet owners without any concerns about plant-based diets were the most likely to feed a plant-based diet to their pet. This may represent the most important consideration for vegan pet owners regarding their decision whether or not to feed a plant-based diet to their pet. Despite most vegans expressing interest in feeding their pet a plant-based diet (Fig 3), the concern about the unnaturalness of the diet may prevent them from doing so. Interestingly, concern regarding the cost of plant-based diets was also positively associated with likelihood of feeding plant-based diets (Table 4). While initially this may seem counter-intuitive, as cost of plant-based diets was predicted to be a potential barrier to some pet owners, it may be that those who do not feed their pet plant-based diets had no concern for the cost of plant-based diets since they were not purchasing them, whereas those who do feed plant-based diets had concern regarding the cost as they were the ones who were affected by the cost of these diets. Most plant-based diets commercially available in the countries best represented in this survey have a high price point, as they are predominantly produced by small, niche companies within the pet food market. It is thus interpreted by the authors that concern for the unnaturalness of plant-based diets for pets is the most predictive factor against feeding a plant-based diet, while a lack of concern about plant-based diets is the most predictive factor in support of the practice.

The findings of this study suggest that more pet owners are interested in feeding a plant-based diet than currently do so, especially those avoiding meat in their own diet. An association between meat avoidance and guilt associated with pet food has been previously documented [27, 40], which would reinforce these results. That being said, the majority of pet owners indicated that they would not feed a plant-based diet, even if one were hypothetically available that met the needs of their pet. This suggests a negative affective response to the concept of feeding plant-based diets to pets [42]. This was anticipated, considering that the majority of people consume an omnivorous diet themselves, and is in agreement with previous studies which have demonstrated reluctance by many people to consider implications of animal consumption or change eating behaviours [67–70]. Additionally, the perceived paucity of evidence supporting plant-based diets for pets, concern of veterinary disapproval, and challenges with availability reported in this survey all likely contribute to an effective case against plant-based pet foods. For those pet owners who indicated that they would consider feeding a plant-based diet if further stipulations were met, the most frequently cited desire was for evidence of nutritional sufficiency. Trends in the pet food industry are certainly driven in part by consumer demands, whereby popular consumer beliefs, and not nutritional requirements, often dictate diet formulation [71, 72]. This suggests that future research regarding the nutritional content of plant-based pet diets, the availability or efficacy of plant-derived nutrients for pets, and the health effects of plant-based diets on pet dogs and cats is warranted from a market demand perspective. Furthermore, predicted population growth and associated increasing demands for protein are placing increasing pressure on the human food system, and the pet food industry may need to adapt in order to avoid competition with the human food supply [16, 73, 74]. As such, regardless of pet owner philosophy or diet, prevalence of plant-based pet feeding practices may increase by necessity. Interest or awareness of this may already be present, which may be an explanation for the number of omnivore and pescetarian pet owners who indicated an interest in feeding a plant-based diet (Fig 3).

As hypothesized, there were differences in the concerns reported by pet owners who avoided meat in their diet and those who did not. This appears to support the findings of psychology studies that have found differences in the consideration for animal wellbeing between vegetarians, vegans, and omnivores [70, 75–77]. These concerns, however, were not consistent throughout the spectrum of meat-avoidance. Significant differences in the number of concerns reported were detected not only between the extreme ends of the diet spectrum (omnivores vs vegans), but also between more similar diets like vegans and vegetarians (Table 3). This is likely attributable to the more ideological lifestyle adopted by most vegans, as opposed to the vegetarian diet [76, 77]. These results are supported by the differences in perspectives regarding animal ethics and empathy towards animals previously documented between vegetarians, vegans and omnivores [21, 75, 76]. As such, the findings of this survey fit with our understanding of the differences between these groups of people in the general population and the described psychology of animal consumption [67, 69, 70]. In published psychology studies, people who reported consumption of meat were found to have less empathy for animals and less concern for animal rights, even amongst those who considered themselves ‘conscientious omnivores’ [78, 79].

It should be noted that, in the present survey, no definitions were provided for the concern categories listed [ie: animal welfare vs animal rights], and thus the distinction between these would have been open to the interpretation of the individual respondent. That being said, the number and type of concerns regarding animal products reported by the vegan and vegetarian groups agrees with past studies examining the ethics and beliefs of vegetarians and vegans [5, 76, 80]. The present data suggests that those choosing to abstain from meat consumption have more concern for the animal from which that meat came from, regardless of whether they are consuming those products themselves or feeding them to their pet. While improvements in animal welfare have been heavily promoted in recent years in response to the demand for ‘humane’ food, it is unlikely that many vegans could be persuaded by these claims due to their moral objection of animal use and not just overt abuse [80]. As such, it is predicted that as the prevalence of veganism increases, the demand for plant-based pet foods will also increase, regardless of the expansion of ‘ethically-raised’ meat in the pet food market [71, 81].

Almost all survey respondents reported concerns regarding plant-based diets for pets. As with concerns regarding animal-based diets, pet owner diet was the most significant factor associated with concern regarding plant-based pet foods (Table 3). Concern for the lack of animal ingredients, and the effect this may have on the nutritional adequacy of the diet and the animal’s health and wellbeing, was the most significant factor. Concern regarding the nutritional adequacy of plant-based pet foods was the most common concern, and was consistently the most reported concern by all pet owners, though this was lower in vegans. This concern is certainly warranted, considering the challenges of formulating a plant-based diet that is nutritionally complete and balanced according to the nutrient profiles published by the Association of American Feed Control Officials and the European Pet Food Industry Federation, based on nutrient requirements established by the National Research Council [82–84]. This is particularly true for carnivorous cats. Obligate carnivores are defined as such due to their unique evolutionary dietary idiosyncrasies resulting in requirements for nutrients not found in plants, such as vitamins A and B12, and taurine [35, 85, 86]. These concerns are not limited to pet owners, but have also been expressed within the scientific community [30, 34, 35]. Few studies have evaluated the nutritional content of plant-based pet foods [34, 35, 87, 88]. Within these studies, the adequacy of the diets varied widely. Furthermore, labelling compliance was determined to be poor [35]. Unfortunately, these phenomena appear to be common within the pet food industry, with multiple accounts of commercial pet foods failing to meet labelling standards, guaranteed analysis, industry recommended nutrient profiles, or containing ingredients other than those listed on the packaging [89–91]. Thus, this does not appear to be an issue exclusive to plant-based diets. Studies evaluating health parameters and nutrient status of dogs or cats fed plant-based diets are also few in number [28, 34, 88]. Clearly, neither the nutritional adequacy of commercially available plant-based pet foods, nor the short- or long-term effects on the physiology and health of pets eating those foods are well defined, contributing to the scepticism reported by pet owners in this current study. These have been identified as areas of future study regarding plant-based pet nutrition.

For those pet owners who reported concern regarding the unhealthiness of meat-based pet foods, it is likely that there is a degree of confounding associated with the distrust of pet food companies and manufacturing process as opposed to the ingredients alone. Due to the survey design, these two ideas were not able to be fully separated, and thus this finding must be interpreted with some caution. Some pet owner comments regarding concerns for animal products reported in the ‘other’ section were regarding the possibility of contamination, the quality of the animal ingredients, or the suspected origin of the product or its ingredients, with China being particularly mentioned by American pet owners. It would thus appear that at least some of the reported concern regarding the unhealthiness of meat-based pet food was not simply the fact that the food contained animal ingredients per se, but the sourcing or processing of those ingredients. This finding, while unquantified in this study, supports other recent reports of distrust in the pet food industry and motivations for feeding non-commercial or alternative diets [92, 93].

While data from previous studies suggested the prevalence of pet ownership amongst vegetarians and vegans may be higher than the generally reported rate of pet ownership, no previous study had reported prevalence of vegetarianism or veganism among pet owners [27, 54]. The results of this study appear to support our expectation that vegetarianism and veganism were higher in pet owners than reported rates in the general public, where reported rates were available (Table 2) [1, 3, 43–53]. Also in agreement with previous studies of the general public, avoidance of meat consumption was also more common in younger age groups than in older (Fig 1), [37, 56, 94]. It warrants consideration, however, that the results of this current study were compared to a number of previous studies which report results from surveys with variable sampling methodologies spanning multiple years. Furthermore, the apparent higher percentage of vegetarians and vegans may also be attributable to the proportion of younger people, aged 21–30 years old, completing the questionnaire (Table 1). To confirm these findings, this study should be repeated with samples of pet owners and non-pet owners in order to directly compare the two populations.

Limitations were present in this study, and results must be interpreted in light of these. Due to the limitation of language, the sample was mostly representative of pet owners in English-speaking countries (Great Britain, the United States of America, Canada, Australia and New Zealand), though there was also a large sample size from Finland. The sample of pet owners responding to the survey was based on self-selection. As such there was risk of sampling bias, which probably accounts for the gender bias detected. Though this was not unexpected, considering the gender-skewed responses to previous studies regarding pet nutrition [92, 95, 96], the strong bias towards female pet owners was even greater than predicted. Considering that females have been reported to have higher empathy towards animals than males and are more likely to avoid eating meat, this may have increased the proportion of meat-avoidance and concerns for animal welfare and/or animal rights reported by survey pet owners when compared with the overall pet owner population [22, 56, 76, 97]. That being said, females are also reportedly more likely to keep pets, so the results may yet be indicative of the general pet owner population [27]. Considering the nature of internet-based surveys, there is also the possibility that the sample acquired in this study was not truly representative of the pet-owning population at large and may have included an increased percentage of vegetarian- and vegan-biased respondents and/or pet owners with particular interest in pet foods. Furthermore, it may be that owners following an alternative or unconventional diet themselves are more likely to have an interest in discussing pet nutrition.

## Conclusion

This study represents the first investigation into the prevalence of meat-avoidance in the pet owner population. The prevalence of vegetarianism and veganism was higher in the pet owner sample than has been reported in the general population, accounting for approximately 12% of pet owners in the sample population. To put that into perspective, in the USA alone, with its population of 325 million [98] and a national a pet-owning rate estimated at 56% [54], there may be as many as 20 million vegetarian and vegan pet owners. Given that the concerns regarding animal-based pet foods reported by vegan and vegetarian pet owners surveyed appear to be the same concerns that they feel regarding animal-products in their own diets, a large number of these pet owners likely desire alternative diets for their pets. Indeed, nearly one-quarter of vegan pet owners reported currently feeding their pets a plant-based diet, while almost half of those who indicated they did not currently do so reported that they would if there were a plant-based diet available which met their standards. For the majority of pet owners interested in feeding a plant-based diet to their pet, the major obstacle was a lack of evidence of nutritional sufficiency. These results suggest a discordance between perceived or real availability of suitable plant-based pet foods and the demand for evidence-based complete and balanced plant-based pet foods. It is clear that an association exists between the diet a pet owner has chosen to follow and the diet they choose to feed their pet. Nutritional sufficiency of most plant-based diets has yet to be demonstrated, while few studies have investigated the short or long-term effects of plant-based diets on pet health. Considering the number of pet owners found to be feeding, or interested in feeding, plant-based diets to pets, and the implications on pet health, nutrition, and the pet food market, more research is warranted regarding plant-based foods for dogs and cats.

## Supporting information

S1 Dataset Questionnaire results. (SAV)
