# Correction: Plant-based (vegan) diets for pets: A survey of pet owner attitudes and feeding practices

**DOI:** 10.1371/journal.pone.0268982

**Published:** 2022-05-19

**Authors:** Sarah A. S. Dodd, Nick J. Cave, Jennifer L. Adolphe, Anna K. Shoveller, Adronie Verbrugghe

After publication of [1], the *PLOS ONE* journal was notified about the omission of the original questionnaire and the corresponding coding key from the Supporting Information. The missing information can be viewed under DOI: https://doi.org/10.5683/SP3/7AOEQS. The files have been assessed by senior members of the journal staff. With this correction, all relevant data are now provided.
